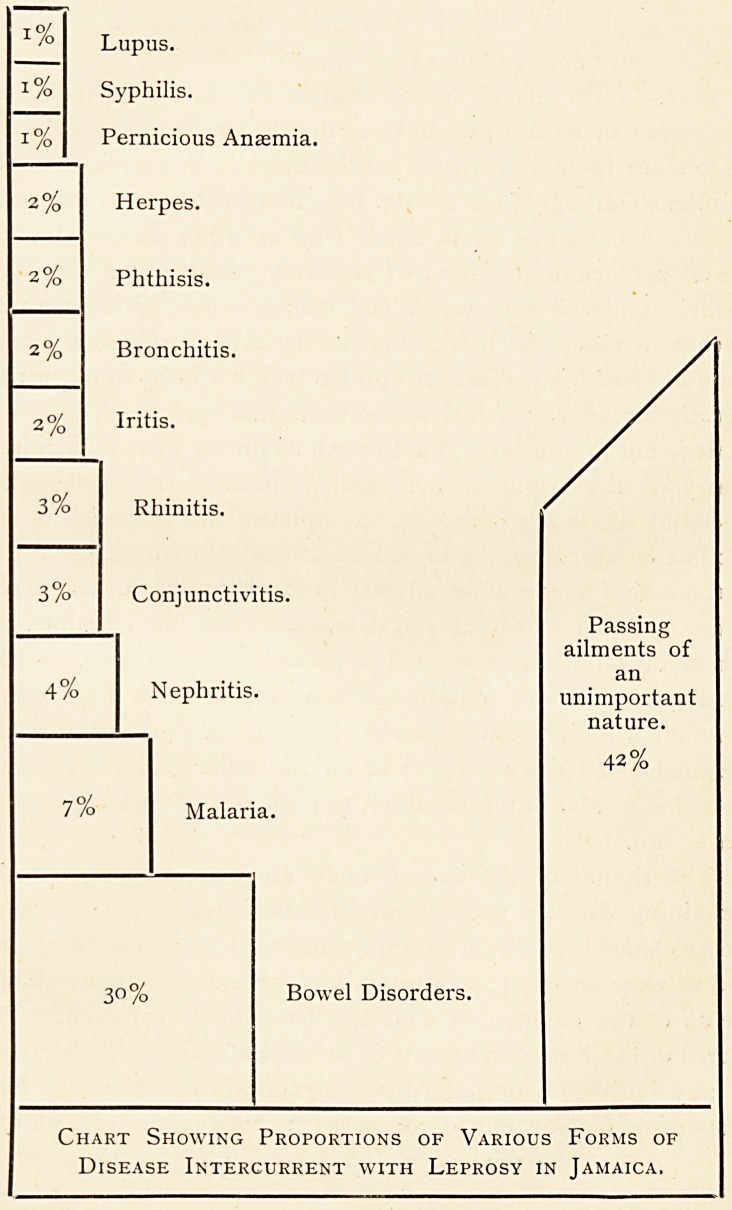# Leprosy in Jamaica
1Read by Mr. Tonkin at a Meeting of the Society on December 9th, 1903.


**Published:** 1904-03

**Authors:** W. D. Neish, T. J. Tonkin

**Affiliations:** Medical Superintendent, Spanish Town Leper House, Jamaica


**Fig. 1.**

*Type of Jamaican Leprosy.*

A nodular case in which the disease is taking an aggressive form, probably in consequence of the existence of some such constitutional weakness as that expressed by the term "strumous."

**Fig. 2.**

*Type of Jamaican Leprosy.*

An instance of the anæsthetic variety of the disease. The patient is upwards of eighty years old, and dates the onset of her disease nearly fifty years back. She has lost the greater part of her extremities and is almost blind, but with the exception of occasional bowel trouble her health is much what would be expected at her age. It is interesting to note that she is a relic of the days of slavery. She was probably somewhere about fourteen years old on that August morning in 1834 when the Act of Emancipation came into force in our West Indian possessions.

### The Bristol Medico-Chirurgical Journal.

*"Scire est nescire, nisi id me*
*Scire alius sciret."*

MARCH, 1904.

### LEPROSY IN JAMAICA.[1]

BY

W. D. NEISH, M.D.

*(Medical Superintendent, Spanish Town Leper House, Jamaica)*

AND

T. J. TONKIN, L.R.C.P., L.R.C.S. Edin.

THERE is a singular appropriateness in reading a paper on "Leprosy in Jamaica" to a Bristol audience. In the first place, since the establishment of the Imperial Direct Mail Service, Jamaica has come to be a dependency more particularly your own, and one therefore in which you might reasonably be expected to feel more than ordinary interest. But there is yet another reason. Men of your own city played in the past no inconsiderable part in introducing the disease into Jamaica. There can be no doubt that leprosy was carried to the New World in general and to Jamaica with the rest in the train of the transatlantic slave trade; and of the part

[1] Read by Mr. Tonkin at a Meeting of the Society on December 9th, 1903.

which Bristol men and Bristol ships took in that domain of enterprise history saves me the trouble of speaking.

The scheme of the paper which I have to read to you this evening is more or less as follows:—In the first place I shall briefly touch in a general way on Jamaica itself; I shall then review the main facts about the disease in the island; and I propose finally to discuss one or two points that are at present of interest. These will include the treatment of the disease by intra-muscular injection of perchloride of mercury, the relation of diet to the disease, and the general question of curability and its bearing on the problem of compulsory segregation.

*Geographical Details.*—It is perhaps not altogether unnecessary, even in Bristol, to begin by saying where Jamaica is. I cannot vouch for the occurrence, but I have heard of gentlemen even in the hallowed precincts of certain rooms in Savile Row looking for it on a map of Eastern Asia. It is not in Eastern Asia. It is not even as so many suppose in the Gulf of Mexico, neither is it in the Atlantic Ocean, but in that adjunct of both called the Carribean Sea. It is one of the Antilles. The island itself is somewhere about 144 miles in length and 47 miles wide. It is a mass of mountains. The main chain runs east and west, dividing the littoral into two almost equal portions. The area of the island is 4,200 square miles, nearly half of which is at various elevations of from 1,000 to over 5,000 feet. All the principal towns are on the seacoast.

It will be seen that in some directions Jamaica offers within a small area widely-varying conditions. The results, however, of nearly thirty years' continuous observation do not show that particular location, geological or climatic conditions, have anything special to do with determining the occurrence of leprosy, the seacoast and the interior, lowlands and high altitudes, clay, limestone and sandy formations having during that time all in the same way provided foci of the disease. The evidence afforded is therefore opposed to the supposition that leprosy is in any way specifically related to any of these circumstances, and in this respect it coincides with results obtained elsewhere.

*General Details.*—For many years past leprosy has been decreasing in Jamaica. Forty years ago there are reported to have been about 800 lepers in the island.[1]

I am able to report that to-day, among a population that since then has nearly doubled itself, there is probably less than a third of that number.

It is Dr. Neish's opinion and also mine that the major part of this decrease is due to the influence of segregation. Since 1878 the island has been provided with a leper asylum. The present institution, of which my collaborator Dr. Neish is chief, is situated at Spanish Town, some fifteen miles from Kingston. For many years past Dr. Mosse, the principal medical officer of the island, has made this home the object of special attention; and at the present I may say for myself that I think it comes as near the ideal leper hospital as is possible where money is as "tight" as it is in Jamaica.

The records of the home give evidence of the decrease of the disease in the island. From 1878 to 1895 they show annual admissions ranging roughly from thirty-nine in the earlier years to fifteen in the later. Up to the latter year, 1895, residence in the home was optional to lepers, there being no compulsory clause. In 1895, however, a Bill[2] was passed in the Legislative Council which had the effect of bringing all pauper lepers into the home, and this measure immediately sent the yearly admissions up to forty once more, but from that time down to the present they have again year by year steadily decreased. It is worthy of note that the decrease has been more regular since the passing of the law than before.

NUMBER OF ADMISSIONS TO THE SPANISH TOWN LEPER HOME FROM 1890 TO 1902.

| Year. | Admissions. | Remarks. |
|---|---|---|
| 1890 | 35 | Decrease irregular. |
| 1891 | 36 | |
| 1892 | 15 | |
| 1893 | 27 | |
| 1894 | 21 | |

[1] In 1872 Dr. Bowerbank gave the following figures to Dr. Gavin Milroy:—

| Years. | Population. | Lepers. |
|---|---|---|
| 1861 | 441,264 | 778 |
| 1871 | 506,154 | 749 |

[2] Law 15, 1896.

PAUPER AND VAGRANT LEPERS COMPELLED TO ENTER.
(Law 15 of 1896.)

| Year. | Admissions. | Remarks. |
|---|---|---|
| 1895–96 ... ... ... | 37 | Years of increased influx due to compulsory law. |
| 1896–97 ... ... ... | 40 | |
| 1897–98 ... ... ... | 38 | |
| 1898 99 ... ... ... | 20 | Years of renewed steady decrease in admissions. |
| 1899–00 ... ... ... | 27 | |
| 1900-01 ... ... ... | 19 | |
| 1901–02 ... ... ... | 9 | |
| 1902–03 ... ... ... | — | |

With regard to the total number of lepers in Jamaica we are not able to give exact figures, as there is no arrangement for enumeration. Just now, however, there are about 110 cases in the home, and as far as Dr. Neish has been able to ascertain, probably somewhere about 150 more at large. This latter number is of course conjectural, still it is not at all likely to be much under the mark. If we give the possibility of its being too small the fullest range, and put the total leper population of the island to-day at 300 individuals, we shall probably be very near the mark. There are now about 750,000 people in Jamaica, so 300 will give a leper proportion of 4 in 10,000. We are sure, if anything, that this overstates the case.

*The Disease Itself.*—The following details with regard to the disease itself are, of course, entirely based on the records of the home and Dr. Neish's personal experience there. Out of the 110 persons at present under supervision, 66 are males and 44 females. The population of Jamaica includes blacks—the great majority; coloured people—that is, mixtures in varying proportions of black and white; coolies, an element imported from the East Indies to work on the sugar and banana plantations; and pure whites. In the leper home these various elements are represented as follows:—One person reputed to be pure white, five coolies, and the rest coloured and black—mostly the last.

The types of the disease common in Jamaica follow the general rules observed elsewhere, the so-called anæsthetic form preponderating. The intercurrent diseases that appear to have special preference for the Jamaican leper are, as usual, those that affect the skin and various surfaces—the conjunctival, pulmonary and intestinal, for example—more or less directly continuous with it. They include conjunctivitis, iritis, rhinitis and ozœna, catarrhal and inflammatory affections of the lungs, bowel disorders and nephritis. The following diagram, which is based on the average state of affairs at the Spanish Town home gives at a glance the proportions in which these various disorders occur:—

Chart Showing Proportions of Various Forms of Disease Intercurrent with Leprosy in Jamaica.

The period over which fatal leprous disease usually extends works out for the Jamaica leper house at ten years for the anæsthetic variety and seven for the tuberculated. The average death-rate in the institution is 10 per cent., and the diseases that most usually terminate life are chronic diarrhœa, chronic nephritis, phthisis, chronic bronchitis, pernicious anæmia and syphilis, probably in the order named.

*Diet.*—With regard to the relation which diet holds to the occurrence of leprosy, we have at the present time to deal with three more or less divergent sets of views. We have, first, the opinion so strongly held by Mr. Hutchinson that fish in a badly preserved condition is in some way or other answerable for the occurrence of the disease; secondly, there is the view that inefficient diet is a powerful and common predisponent to the disease, a view which I hold myself; and, thirdly, we have a large body of expressions of opinion which appear to mean that dietetic factors are of little or no importance whatsoever.

It is not my purpose now to push again my own particular [1] theory of the origin of the predisposition of the disease, but I should like to say that in my opinion the influence of the bacillus of leprosy on the human organism is broadly comparable to the action of any other adverse influence on any other object. It is a question of attack and defence. Take, for example, the action of wind and rain on the plaster of a building. If the plaster be well and soundly made we may expect that, within reasonable limits, it will sustain little or no damage from the inclemency of the weather; if, on the other hand, it is made of bad stuff and ill put together, crumbling and leakage may be looked for.

The tissues of the human body are composed of material containing definite proportions of certain chemical elements. These elements are built up into complex organic combinations, and as wear and tear are always in progress, constant reinforcements of the component elements have to be supplied. The source of these reinforcements is the diet, and if the food supply of any individual or race does not contain a sufficiently high proportion of any one or other of the essential elements, a

[1] *Med.-Chir. Tr.*, 1902, lxxxv. 145.

weakness of the resulting structure directly proportionate to the extent and importance of the element lacking will be the inevitable result. The weakness will show itself in various ways, among others as lowered resistance to the action of morbid influences; and as the ill-made plaster of the building yields readily to stress of weather, so will the tissues of a body so unfortunately constituted give way more easily to the attacks of their peculiar adversaries than those of one more properly and completely nourished.

I do not wish to press the point further at the moment. But it is an incontestable fact that all the leper areas of the world coincide with areas inhabited by peoples whose national diets, show and show in many cases very strongly, a marked and definite chemical inefficiency, namely a shortage of nitrogen; that historical instances can be cited to show that the disease has in most cases slowly receded before improving dietetic conditions; and that to-day a relation between cheaper and better food and decreasing leper rates can often be clearly demonstrated. To a limited extent Jamaica is an instance of the last. The exact place I would give to the diet of the Jamaican of to-day among the diets of the world is that of an inefficient diet that is steadily improving.

The poor class Jamaican is, however, still vegetarian to an extreme degree. This is capable of very clear proof. Dr. Neish is surgeon to the police depôt, where the recruits for the Kingston and general island force are trained. When negroes are brought in fresh from the mountain districts it is necessary to put them on to the regular rations of the force, which include one pound of uncooked meat a day, gradually and with the greatest care. They are at first totally unable to digest the meat, and unless precautions are strictly observed it acts as an irritant, and violent attacks of pain, sickness and diarrhœa result.

The diet that such men as these have been taken from is a bare one. At least half—perhaps it will not be too much to say three-quarters—of the population of the island still live almost exclusively on articles among which the starchy roots of tuberous plants figure largely. "Bread-kind" the natives call them.

Yams (*Dioscorea sativa* and *aculeata*), sweet potatoes (*Ipomœa batatas* and *Batatas edulis*), eddoes (*Colocasia antiquorum*) and bread-fruit (*Artocarpus incisa*) are the chief of these. Fruits, such as mangoes, star-apples, oranges, and to a certain extent bananas, are also freely eaten when in season, but the starchy tuber in general and the yam in particular may be taken as the basis of the diet.

The average daily round is much as follows:—

First thing in the morning a hot drink is made by infusing such herbs as cow-foot, lemon grass or lime leaves in water. This is sweetened with sugar and called "tea." On the coast where bread is obtainable it is occasionally eaten at this meal; but up-country, where even unleavened bread is scarce, a piece of the stale boiled yam of the day before takes its place. At midday a pot is boiled containing yams, bread-fruit or any other available "bread-kind," flavoured with an ounce or two of shad (a kind of herring), or mackerel, or of that particular brand of salt cod that is, I believe, known all over the British West Indies as "Halifax Mutton." This meal is eaten about one o'clock. At night, after sundown, another "pot," a big one this time, is put on, in which the eternal "bread-kind"—yams, eddoes, sweet potatoes, as the case may be—again flavoured with a microscopic proportion of "salt-ting," as the salt fish or salt beef element is called, is once more boiled to a soup, the eating of which closes the dietetic round of the day.

The amount of starchy tubers eaten by the ordinary man in the course of the meals of an ordinary day ranges from one to three pounds, that of the more highly nitrogenised elements averages under two ounces.

Things are improving, however, in this direction in Jamaica. There is evidence to show that as a whole the food resources of the people are gradually widening. Cattle have been and are being introduced into the island; an agricultural society has been working for years past towards the improvement of the various indigenous crops and the introduction of others suitable; peasant proprietorship is extending, and these changes mean to Jamaica what all ameliorative changes mean for all races they may affect—in the first place more abundant and better food. Leprosy is emphatically a disease of the ill-nourished classes; improve the circumstances of a race, and you decrease the numerical strength of those classes. The circumstances of Jamaica have been very radically improved during late years, and though it may not be capable of direct proof, there is little doubt that this improvement has not been without its influence in assisting to determine the regular yearly lessening of the amount of disease in the island.

From the question of diet in general we may conveniently turn to that particular article on which so much discussion has lately taken place.

For many years past Mr. Hutchinson has supported with the whole weight of his great experience and influence the theory that leprosy is a disease the incidence of which is in some way determined by the use of unsound fish as an article of diet; and it was suggested to Dr. Neish and myself that we should give special prominence to details with regard to the consumption of fish in the island, with a view of determining the degree of connection between it and the disease. We are afraid Jamaica cannot be relied upon to supply any very definite evidence either for or against the fish theory. The habits of the people with regard to this matter are universal. We have not in Jamaica, as in India, a large number of clearly-defined classes whose customs vary widely and more or less rigidly, nor have we districts that are inaccessible to fish. There is but one class, generally speaking, in the island of Jamaica, and that class eats fish: one district only, and to every part of that district, on the coast and up the country, fish penetrates. That the fish eaten is small in amount has no bearing on the question as at present discussed; it is universally eaten, and that circumstance divests the situation of any special interest from the point of view of those who oppose the fish theory. Nevertheless, I did my best to discover from the figures obtainable what variations in the consumption of fish had taken place during late years. Leprosy has steadily decreased during the last thirty years. The question to be decided was, what movement in the consumption of fish, whether increase or decrease, was during that period to be set against it. With a view to doing this

I passed in review both qualitatively and quantitatively the fish imports of the last thirty years. The figures that resulted were more or less neutral. It does not appear that Jamaica has altered its habits in the matter of fish even fractionally during the period under consideration. In the five years from 1873 to 1877 inclusive the total amount of all kinds of fish imported into the island was a little over 95,000,000 pounds weight. During the last five years, from 1899 to 1903 inclusive, it has been a little more than 122,000,000. These amounts and the rate of increase during the years that intervene agree almost exactly with the course taken by the population, which has increased during that period from 570,000 odd in 1881 to somewhere about 750,000 the year before last. It will be seen these figures have but little active bearing one way or the other. All that we can safely say with regard to fish in Jamaica is, that in small amounts the commodity is universally consumed by a population that includes to-day 300 or less lepers among 750,000 persons, and that though the disease has decreased by two-thirds during the last thirty years while the general population has largely increased, the fish-eating habits of the people, and the type and quality of the fish consumed, appear to remain at the close of that period the same as they were at the beginning.

*Treatment.*—With regard to the treatment of leprosy generally and in its widest sense, the record of the Spanish Town home is a good one. But before I go on to speak of routine treatment and its results I would like to give you some details of the use of one particular remedial measure, namely the intra-muscular injection of perchloride of mercury. There seems to be more unanimity among leprologists with regard to the value of this particular remedy than with reference to any other; and it was suggested to me by Mr. Pernet that as one of us (Dr. Neish) had made extensive trials of it, we should embody some report of the results in this paper. In fighting such a disease as leprosy every fresh-proven weapon is an added advantage, and I am pleased to say that Dr. Neish regards the mercurial treatment as distinctly valuable. Starting in 1899, he has now put about 100 specially-selected patients through the mercurial mill, with favourable results in almost every case. For several reasons Dr. Neish prefers not to use an oily vehicle for the remedy. Perchloride of mercury and common salt, of each a quarter of a grain dissolved in 20 minims of distilled water, is the preparation he thinks most suitable. This is injected through a platinum and iridium needle deep into the muscles of the back or buttock. This injection, which is made twice a week, gives great pain, and causes a lump to rise as hard as a stone and about as big as a pigeon's egg. The pain wears off in from two to four hours, the swelling disappears in the course of a week, and the next injection but one can, if desired, be put in or about the same spot.

Out of the hundred cases treated in this way by Dr. Neish, in only one was any untoward result observed. In this case—a woman—profuse salivation was induced, and the treatment was consequently dropped. In all the others the results have been favourable. Ulcerations have healed or been markedly improved, tuberculations have shrunk, pain has been lessened, the mental condition made brighter, and even in some cases a certain extent of surface sensation has seemed to have been re-established. It is now nearly four years since the treatment was first tried, and the improvement in health brought about in those dealt with then has in almost every case been maintained. That the improvement is one that is evident to the patients themselves seems clear, for if they did not derive distinct benefit from the treatment it is doubtful if they would put up with the pain it causes them. It is worthy of notice that out of the thousands of injections that have been made at the Spanish Town home not one has resulted in an abscess.

We are glad to be able to give so promising an account of this method of treatment. and we think there is no doubt that in suitable cases—that is, those of reasonable strength of body and free from grave organic disease or cachexia—it is a most valuable adjuvant, perhaps the most valuable we have, in the struggle with the disease. More than an adjuvant, however, it can probably never be. I think both Dr. Neish and I agree that in treating leprosy the only method of general application is to act on the assumption that the bacillus of the disease is not of sufficient virulence to maintain its footing in the face of the resistance offered by the tissues of a well-nourished individual under ordinary conditions and in a normal state of health. It is imperative, therefore, in every instance to aim first at the improvement of the health of the person attacked, and to relegate to a secondary place the extermination of the attacking organism.

This is the line adopted by Dr. Neish at the Spanish Town home. Each case is treated on its own special requirements, and the effort is always towards upbuilding. Cleanliness is insisted on. Every patient is compelled to bathe in the large concrete baths provided by the institution at least three times a week, and ulcers are dressed twice daily.

The mental side of the individual is attended to. Books are provided for those who can read, and as many illustrated papers and magazines as can be obtained for all. For those that can play, outfits for cricket and other games are provided. For the evenings there is a lecture-room in which plays, readings and concerts are given. With the exception of those unfit for any form of occupation, none are allowed to remain idle. Light work is found for all, and an interest is given to it by a system of bounties. For the children there is a school, and one of the most interesting of my recollections of the home is that of the dominie—an aged, white-haired, horn-spectacled negro-leper ringing his bell at the schoolroom door, while from various quarters sundry little limbs of night came shuffling up, more or less reluctantly, in answer to the sound.

But it is with the dietary that we come to the main weapon in the armoury of the Spanish Town home. The dietary of the institution is a liberal one. I append a diet sheet [1] to this paper, and it will be seen that instead of getting yam, with a bit of salt fish to flavour it, as his staple food, the leper, after entering the home, finds himself face to face with a menu that includes, as well as his national foods, bread, milk, meat and peas. There can be no reasonable doubt that the improvement that takes place in the condition of so many of the inmates almost immediately on reception is due to the change for the better in their

[1] Appendix I.

diet, and due to that almost solely. No reasonable person watching case after case pass in and at once pick up without any other than general attention could come to any other conclusion.

The laws of the island do not provide for the compulsory segregation of any but pauper and vagrant lepers; many, therefore, do not come to the home till they are so broken up by the disease as to be unable to do anything more for themselves. It is Dr. Neish's firm conviction that if he could get these cases earlier he could in almost all instances, except where the presence of grave constitutional vice renders the progress of the disease unavoidable, prevent the occurrence of the paralyses, ulcerations and mutilations that have come to be regarded as the almost necessary outcome of the disease. In other words, that he could by careful personal attention, dietetic and hygienic measures vastly modify and often cure the disorder, and in this matter I entirely agree with him. Feed your leper, and you break the back of his disease.

These convictions are amply supported by the records of the home. Appended to this paper you will find the report of a case [1] exactly as it stands on the books of the institution. It is interesting evidence on this point. When first admitted the patient was 53 years of age, and had been leprous many years. He was in a condition of advanced anæsthesia. He had lost all his fingers and toes, was much emaciated, and had a large plantar ulcer. He entered the home in February, 1899, and was placed at once on the liberal dietary of the institution. No other measures—no mercurial, chaulmoogra, or other treatment—were adopted; rest, cleanliness and good food were allowed full and sole play. By January, 1900, after a lapse of eleven months, the plantar ulcer had filled up, and the man's condition is reported as vastly improved; while thirteen months later still, in February, 1901—two years, that is, after his first reception—finding the good health maintained and all ulcerations remaining soundly healed, Dr. Neish recommended the case to His Excellency the Governor as one for discharge, and the man was accordingly sent home in May of the same year.

[1] Appendix II.

That was May, 1901. It is now December, 1903, and it is of interest to know that there has been no return of ulceration, that the man's general health is still good, and that to-day he is doing something towards earning his living.

I am not throwing stress on this matter of recovery with the idea that I am bringing anything new to the notice of those intimately associated with leprosy. Doubtless, indeed, many who are not in close touch with the disorder are aware of the facts on this point. Nevertheless, a notion that the disease is incurable is widely spread among members of our profession. A glaring statement to that effect finds a place on the pages of the latest edition of at least one educational handbook of medicine that I could mention, and it is with a view to doing what I can towards the correction of this idea that I am making these remarks. Leprosy is a disorder of which every grade exists. In every endemic area where one person suffers from a severe grade many contract slighter degrees from which they may recover without outside help, and even probably in some cases without recognising the condition. And among those who take the disease more severely, improved surroundings and more wholesome diet may be relied upon to very greatly improve, and in an appreciable proportion of the cases cure, the disorder. The process is slow, but I have not seen one nor two, but scores upon scores of cases in which its success had been beyond a doubt, and as a result I would put "incurable" out of the list of adjectives that may with propriety be applied to leprosy.

The bearing of a true appreciation of this matter on a course to be pursued in any given case, or the policy of any given institution, is simple but important.

In the past, at any rate, many so-called leper sanatoria have been merely prisons for the confinement of persons supposed to be dangerous to their respective communities. Once the gates of such institutions closed on a leper, for all practical purposes that leper ceased to be. Such a thing as providing for recovery was not thought of, and the deprivation of hope robbed the victim of the system of much of the energy that should have helped him, if not to recovery, at least back on the way to it.

The home at Spanish Town is no penal settlement of this kind. Dr. Neish preaches to his patients the gospel of hope; and he has been able to show them that their hope is not without foundation, for in spite of the extreme caution which, in view of the present state of public feeling, has to be exercised in putting opinions of this kind into effective operation, he has been able during the last four years to discharge fifteen of his patients, not one of whom has given him any cause to regret his decision.

*Summary.*—Leprosy is decreasing in Jamaica. During the last thirty to forty years the number of lepers has been reduced by about two-thirds; the proportion to-day does not exceed four out of every ten thousand inhabitants. The island is provided with an admirable leper home, and the decrease in the amount of the disease is probably largely due to the influence of this institution.

In common with many other parts of the world that are leper-smitten, the diet of the native Jamaicans is markedly deficient in nitrogenous elements. The average native comes so rarely in contact with any appreciable quantity of highly nitrogenised food stuff, that an ordinary beef-steak acts on him as an intestinal irritant. The general condition of the people, and with it the diet, are, however, steadily improving.

Fish is universally consumed by the Jamaicans; but from the data obtainable it is not possible to make any definite statement as to whether or not it is in any way causally related to leprosy. Such small balance of evidence as there is would, however, appear to indicate that no such relation exists.

The treatment of the disease by the intra-muscular injection of perchloride of mercury has been extensively tried in Jamaica, and there is little doubt of its value as an adjuvant to good feeding and careful general attendance. A hundred selected cases have been treated in this way with benefit, sometimes very considerable, in almost every case.

As to general treatment, both the writers of the paper are convinced that in leprosy the line most generally applicable is that which improves the bodily condition of the person affected, and their experience leads them to believe that an improved diet is the most powerful agent in this direction, and the one most requently required.

They both also regard leprosy as a disease that is in many nstances capable of sound cure.

### APPENDIX I.

### DIET SCALE.

*Institution Diet.*

6.45 a.m. Coffee, ground, ½ oz., or tea made of native herbs, ½ pint; milk, 1 oz.; sugar, 1 oz.; bread, 4 ozs.

*Breakfast.*

11.0 a.m. Monday, Thursday and Saturday—Fresh beef or goat mutton, 4 ozs.; barley, ¾ oz.; flour, wheaten, 2 ozs.; yam, 4 ozs.; half a lime; salt, 1 dram.

9.30 a.m. Tuesday and Friday—Bread, 4 ozs.; butter, ½ oz. Wednesday and Sunday—Porridge, consisting of cornmeal, 4 ozs.; milk, 5 ozs.; sugar, 1 oz.; bread, 4 ozs. (On Sunday no bread will be issued for breakfast.)

*Lunch.*

12.30 p.m. Sunday, Tuesday, Thursday and Friday—Bread, 4 ozs.; sugar, 1½ oz. Monday, Wednesday and Saturday—Bread, 4 ozs.; fruits in season. (On Wednesday no bread will be issued for lunch.)

*Dinner.*

4.30 p.m. Monday, Wednesday, Friday and Saturday—Fresh beef, 4 ozs.; vegetables, 2 ozs.; yams, 12 ozs., or rice, 6 ozs.; half a lime; salt, 1 dram. Tuesday and Thursday—Yam, 12 ozs.; salt fish, 6 ozs.; cocoanut oil, ½ oz.

3.30 p.m. Sunday—Red peas, 4 ozs.; yam, 8 ozs.; salt beef, 4 ozs. (6 ozs. of rice may he given to coolies in lieu of yam if they desire it.)

*Hospital Diet.*

7 a.m. Arrowroot, 1½ oz.; sugar. 1½ oz.; milk, 5 ozs.

9 a.m. Sago, 1½ oz.; sugar, 1½ oz.; milk, 5 ozs.

5 p.m. Arrowroot, 1½ oz.; sugar, 1½ oz.; milk, 5 ozs.

Stimulants: Whisky, port wine, gin.

Extras: Fresh beef, ½ lb.; eggs, 1 or 2; butter, 1 oz.; milk, 10 ozs.; rice, 3 ozs.; bread, 4 ozs.; biscuits, 4 ozs.; coffee or herb tea, half-pint; condensed milk.

*Note.*—Extras are only allowed at the discretion of the medical superintendent.

### APPENDIX II.

JAMAICA LEPER ASYLUM. CASE BOOK D, NO. 12.

P—— G——, male, aged 53 years, a brown labourer, was admitted on the 25th February, 1899, from Cornwall, St. Elizabeth. He thinks the disease was acquired at Lacovia, in the same parish.

*Family History.*—Both parents dead; cause of death unknown. Has two brothers and two sisters, in good health and free from the disease. As far as he knows, no member of his family is afflicted with leprotic disease.

*Personal History.*—Was born at Cornwall, St. Elizabeth. Has been vaccinated; is unmarried, but is the father of one child. Has always enjoyed good health until the present disease developed many years ago, the first symptoms being painful joints, numbness and cramps, and later on ulceration of fingers and toes. Has always resided in Jamaica.

*Present Condition.*—This is an advanced case of anæsthetic leprosy. Patient much emaciated. There is a large superficial ulcer on the sole of the right foot. The toes and fingers of all the extremities are absent.

*January, 1900.*—This man's condition has vastly improved. The ulcerations have entirely healed. The general health is improved.

*February, 1901.*—The good health of this inmate is maintained. No ulcers exist. Recommended for discharge under Section 9.

Discharged by order of His Excellency the Governor on the 7th May, 1901.

## Figures and Tables

**Fig. 1. f1:**
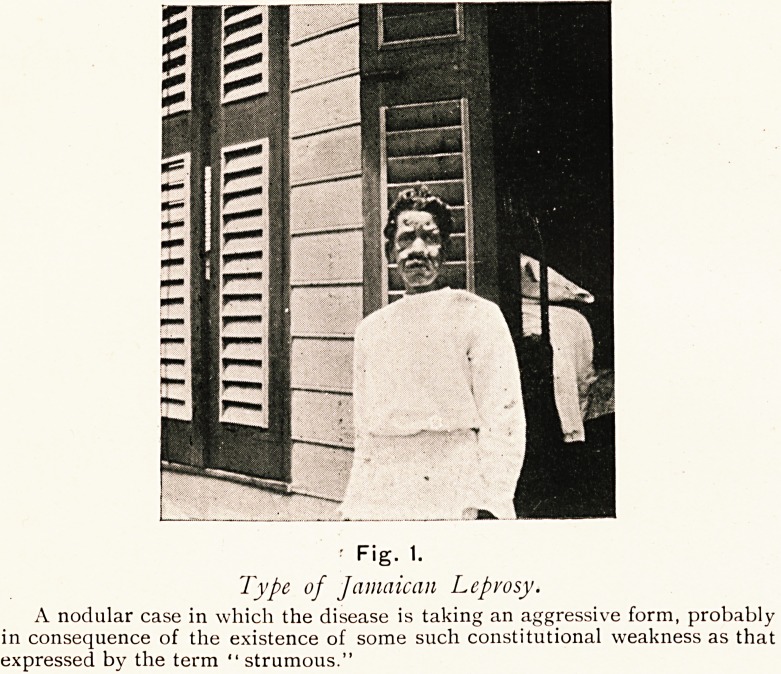


**Fig. 2. f2:**
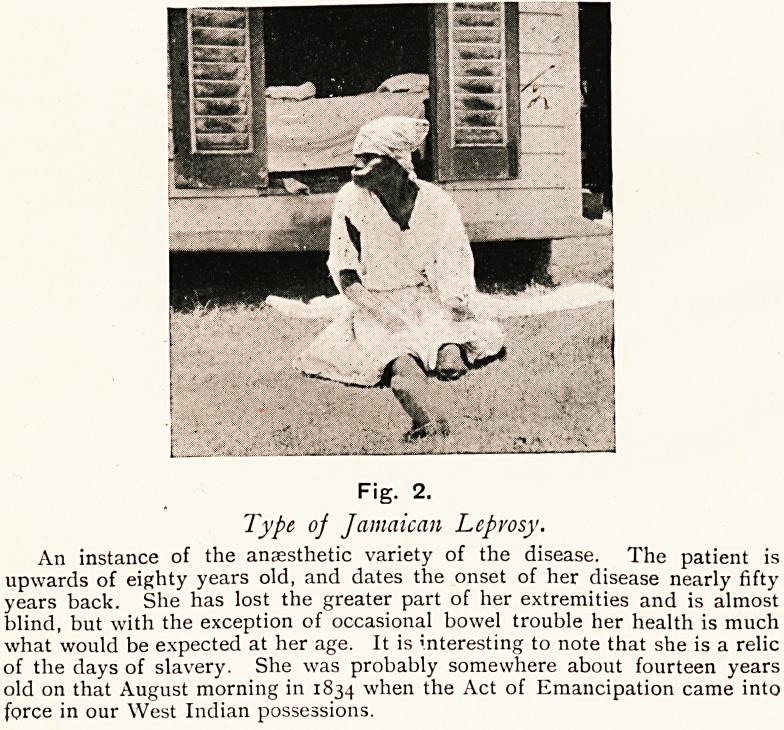


**Figure f3:**